# Self-harm in 5-to-24 year olds: Retrospective examination of hospital presentations to emergency departments in New South Wales, Australia, 2012 to 2020

**DOI:** 10.1371/journal.pone.0289877

**Published:** 2023-08-10

**Authors:** Michelle Torok, Alexander C. R. Burnett, Lauren McGillivray, Jiahui Qian, Daniel Z. Q. Gan, Rachel Baffsky, Quincy Wong

**Affiliations:** 1 Black Dog Institute, University of NSW, Sydney, Australia; 2 School of Population Health, University of NSW, Sydney, Australia; 3 School of Psychology, Western Sydney University, Sydney, Australia; University of Bern: Universitat Bern, SWITZERLAND

## Abstract

There is some evidence that self-harm presentations in children and young people have increased over the past decade, yet there are few up-to-date studies examining these trends. This study aims to describe trends in the rates and severity of emergency department self-harm presentations for youth aged 5–24 years in New South Wales, Australia between 1 January 2012 and 31 December 2020. We analysed self-harm hospital presentations using join point analysis to compare quarterly growth in rates and urgency of presentation since 2012 by age group and sex. Binomial logistic modelling was used to identify risks for re-presentation for self-harm, including age group, sex, country of birth, mode of arrival, inpatient status, triage category, rurality, and socio-economic disadvantage. In total, 83,111 self-harm presentations for 51,181 persons were analysed. Overall rates of self-harm among those aged 5–24 years increased by 2.4% (*p* < .001) per quarter in females and 1.6% (*p* < .001) per quarter in males, with statistically significant average quarterly increases observed across all age groups. Overall and age-specific self-harm triage urgency rates increased statistically significantly for potentially serious, and potentially- and immediately life-threatening categories. A higher likelihood of re-presentation to any emergency department for self-harm was associated with younger age, female, residing in a regional area, arriving by ambulance, admitted as an in-patient, and a more severe index self-harm presentation. Hospital self-harm presentations have been growing steadily over the past decade, with the greatest growth in the youngest people. Understanding the reasons for these sustained upward trends is a priority for suicide prevention.

## Introduction

Self-harm (self-poisoning, self-injury with, or without, intent to die) by young people is a major public health concern, for this behaviour not only increases the risk of premature mortality due to suicide but also contributes significantly to the disability burden [1,2]. While around 10% of adolescents in the community self-harm [3,4], it is estimated that only one in eight will present to a hospital for self-harm injuries and/or suicidal distress [1,3,5,6]. Though individuals who present to hospital may only account for a small proportion of all young people who self-harm, their risk of suicide is considerably higher [1,7–9]. Ongoing efforts to improve current understandings of these ‘higher risk’ young people and the nature of their self-harm presentations is critical to developing appropriate crisis management plans and interventions for hospital settings, to prevent suicide.

Globally, numerous epidemiologic studies show a decrease, and levelling off, of rates of hospital-treated self-harm among adolescents and young adults up until 2010 [10–15]. Since 2011, however, there has been a concerning reversal of these trends, with numerous studies demonstrating strong upward growth in the rates of hospital-presenting self-harm by young people [10–14]. For example, in the USA, paediatric hospital admissions for suicidal ideation and self-harm have doubled between 2008 and 2015 [13] and significant increases in serious self-poisoning presentations were observed among 10–18 year olds from 2011 to 2018 [14]. In Canada, self-harm hospital presentations among 13–17 year olds increased by 135% from 2009 to 2017 [12]. A recent Australian study [16] found that suicide-related presentations by young people (ages 10–24 years) increased by 8.4% per annum between 2015 and February 2020 (pre-coronavirus [COVID] pandemic), and then growth trends more than doubled (to 19.2% per annum) from March 2020 into 2021.

Adolescence, between ages 14 and 19, is typically the peak risk period for hospital-treated self-harm [5,9,15], and from ages 11 onwards, females are between two and four times more likely to present to an emergency department (ED) for self-harm than age-comparable males [3–6,10,17]. It is unsurprising then that current knowledge about youth self-harm is derived almost exclusively from adolescent populations. Children younger than 12 are rarely represented in self-harm research, primarily due to concerns that intentional self-harm behaviour may not be reliably identified. However, research shows that self-harm can be identified in children as young as six [18]. Where studies have sought to include children, they have shown that self-harm is increasingly affecting those under 12 years of age. For example, Griffin et al [10] found that between 2007 and 2016 rates of self-harm increased overall by 22%, with the largest increases noted among 10–14 year olds (75%). Sara et al [16] found that children aged 10–12 experienced the highest annual growth in self-harm presentations to hospital following the onset of the pandemic, compared with adolescents and young adults. In the UK, there is evidence that community-occurring self-harm among 12–14 year olds has a similar incidence to those aged 15–17 years [5] in both females and males, highlighting that self-harm is not simply a problem of adolescence. More attention to understanding the onset and occurrence of self-harm in children and younger adolescents is needed, as is identifying modifiable risks, triggers, and biopsychosocial processes that lead to this behaviour in children.

Understanding patterns of self-harm is highly relevant for developing and strengthening prevention, early intervention, and crisis management responses for young people, yet the notable knowledge gaps around children and timeliness of data (with many studies reporting data from 2016 or earlier, e.g., [3,5,6,18–20] limit the potential for findings to shape effective responses to this issue. In this study we sought to establish temporal trends in both the rates and severity of hospital-presenting self-harm among young people aged 5–24 years, for the period of January 2012 to December 2020, using hospital administrative data collections for NSW, Australia. We also examined the frequency of ED re-presentation, and whether sociodemographic and clinical characteristics explained ED representations for self-harm. The overall aim was to describe the changing nature of hospital-presenting self-harm in children compared to adolescents and early adults to assist schools, health systems, and community organisations who are responsible for supporting vulnerable youth.

## Methods

### Design and setting

This retrospective study used routinely collected and aggregated hospital administrative data of individuals aged 5–24 years who presented to a New South Wales (NSW) Emergency Department (ED) for self-harm during the period of 1 January 2012 to 31 December 2020 (inclusive). Ethical approval for this study was granted by the NSW Population and Health Services Research Ethics Committee (2020/ETH01527).

### Data sources

De-identified patient level data was obtained from the NSW Health Emergency Department Data Collection (EDDC), via the NSW Centre for Health Record Linkage. The EDDC is comprised of routinely collected administrative and clinical data for presentations to all public hospital EDs in NSW. Any records from EDs with incomplete or missing diagnostic information on self-harm were excluded from the current study. The data captured in the EDDC includes demographic information, dates and times of care, urgency (triage) codes, and diagnostic or problem codes recorded in Systemized Nomenclature of Medicine (SNOMED) terminology. In recognition of the limited diagnostic coding available in the EDDC relating to mental health presentations, a study known as the Mental Health Living Longer (MHHL) program [21], was established in 2019 by NSW Health to improve diagnostic sensitivity. To enhance sensitivity, MHHL custodians examine (and code) an unstructured free text field relating to the presenting problem for the ED visit. The current study used EDDC data extracted from the MHHL, and a binary variable (self-harm: yes/no) was created by the MHHL custodians to identify intentional self-harm presentations. Records were excluded where patients were dead on arrival, transferred in from another hospital, or were planned re-presentations to ED.

For rate calculations, data on the underlying NSW population aged 5–24 years were extracted using the Australian Bureau of Statistics (ABS) Census 2016 TableBuilder [22] (representing the approximate midpoint of this study).

### Identification of self-harm presentations

A hospital presentation for self-harm was confirmed if the EDDC record included an International Classification of Diseases, tenth revision, Australian modification (ICD-10-AM) code in the range of X60 –X84, or Y87.0 (intentional self-harm) or the MHHL binary ‘intent’ self-harm variable (self-harm, yes/no). Diagnostic recording in the EDDC principal ED diagnosis field is recorded by treating clinicians using either SNOMED-CT, ICD-10-AM, or the International Classification of Diseases, ninth revision, clinical modification. To reduce variation in diagnostic recording, SNOMED-CT diagnoses for intentional self-harm were converted to equivalent ICD-10-AM codes, using mapping tables provided by NSW Health for analyses. Where the ED principal diagnosis field was allocated to a specific physical injury or mental health condition and the text field recorded at triage indicated intentional self-harm, indicated by the MHHL data, the MHHL binary variable for intentional self-harm was utilised to identify additional presentations. In this study, ‘self-harm’ includes all incidents where clinicians or hospital coders determined that the injury or poisoning was intentionally inflicted, meaning that presentations with, and without, suicidal intent are included.

### Outcomes and risk factors

The primary outcome was the quarterly rate of ED presentations for self-harm per 100,000, calculated overall for NSW residents aged 5–24 years as well as by age groups (5–12; 13–17; 18–24), sex, and triage urgency category (described below). A secondary outcome was the frequency of, and time to, re-presentation to the ED for self-harm, defined as re-presentation to ED from the first episode of self-harm at the person level occurring during the study period.

A limited set of demographic and social characteristics were assessed as potential risk factors for the primary and secondary outcomes. Age, assigned sex at birth (male, female), country of birth (Australia, other), mode of arrival (ambulance vehicle arrival, other mode of arrival), and in-patient admission status (yes, no) were obtained for each presentation. The 2016 Australian Statistical Geography Standard (ASGS) Accessibility and Remoteness Index (ARIA+) and ABS Index of Relative Socio-economic Disadvantage (IRSD) were obtained for the persons NSW Statistical Area Level 2 (SA2) of residence [23]. Persons residing outside of NSW at the time of presentation were excluded. ARIA levels were classified into three areas of remoteness (major cities, inner or outer regional, and remote or very remote). IRSD levels were classified into quintiles. The Australasian Triage Scale [24] was used to define presentation urgency and were classified according to four threat levels: ‘immediately life-threatening’: triage categories 1 and 2; ‘potentially life-threatening’: triage category 3; ‘potentially serious’: triage category 4; ‘less urgent’: triage category 5. Re-presentation status was classified as ’no re-presentation (single event)’ where a person had no subsequent admissions for self-harm during the study period and ‘multiple presentations’ where a person had at least one subsequent self-harm admission following the index episode during the study period. Additional person level presentations that were not related to self-harm were excluded.

### Statistical analysis

Descriptive statistics were used to calculate rates and characteristics of ED presentations by sex, age group, country of birth, remoteness, socio-economic quintile, year, and triage urgency using ABS Census 2016 NSW populations for persons aged 5–24 years as the denominator. Trends of quarterly ED self-harm presentations by age group and sex were estimated for the period 2012–2020 using joinpoint regression analysis [25]. Joinpoint regression was also used to estimate quarterly trends in presentation urgency (triage) categories for the period 2012–2020. Each joinpoint (if any) indicates a significant change in the slope, suggesting a change in trend. The following parameter settings were specified in the models fit in the joinpoint analyses. Random errors were assumed to be heteroscedastic. The grid search method with 0 grid points was chosen to search for the location of each quarterly joinpoint. To determine the optimal number of join points to be tested within each model, Monte Carlo simulation was used with the number of permutations set to 4,499, to calculate the permutation significance value of each joinpoint hypothesis test. For each detected trend, the quarterly percentage change (QPC) was estimated. The significance of the trend change was tested at *p* < 0.05 with reference to the two-sided Student’s t-distribution. Long-term trends over the entire study period are average quarterly percentage changes (AQPC) and were estimated as the weighted average of the short-term QPCs.

The likelihood of re-presentation for self-harm was examined between groups (single presentation vs multiple presentations) using binomial logistic regression analysis. The independent variables included age group, sex, country of birth, remoteness, socio-economic quintile, mode of arrival to ED, in-patient status, and presentation urgency (triage) category. For the multiple presentation group, sociodemographic and clinical characteristics at the index presentation were used. All variables were entered simultaneously into the model, with odds ratios (ORs) and their 95% confidence intervals (95% CIs) reported.

Statistical analyses were performed using SAS 9.4 [26]. Joinpoint regressions were performed using the Joinpoint Regression Program 4.9.1.0 obtained from the Statistical Research and Applications Branch of the National Cancer Institute [27]. Event-based data is used in all analyses, except for the likelihood of re-presentation analysis where person-based data is used.

## Results

### Rates of ED self-harm presentations

In NSW from 2012 to 2020, there were 83,111 presentations for 51,181 persons aged 5–24 years to EDs for self-harm. In total, 52.5%, 44.6%, and 2.9% of presentations occurred among those aged 18–24, 13–17, and 5–12 years, respectively. In 2012, the overall presentation rate was 371.4 per 100,000 which increased by 68% to 624.2 per 100,000 in 2020.

Females were over-represented in these data, accounting for 78.1%, 63.8%, and 63.2% of presentations in those aged 13–17, 5–12, and 18 24 years, respectively. The overall rates by sex and age were highest among females aged 13–17 years, at 3.75 times higher than age comparable males. The highest self-harm rates among males were observed in the 18–24 year age group. Rates varied by remoteness, with young people in regional and remote areas being more likely to present to an ED for self-harm than those residing in major cities. Rates also varied by socio-economic disadvantage, with the highest rates among the second most disadvantaged quintile for females and among the most disadvantaged for males.

### Time trends in rates of ED self-harm presentations

**Fig 1:**
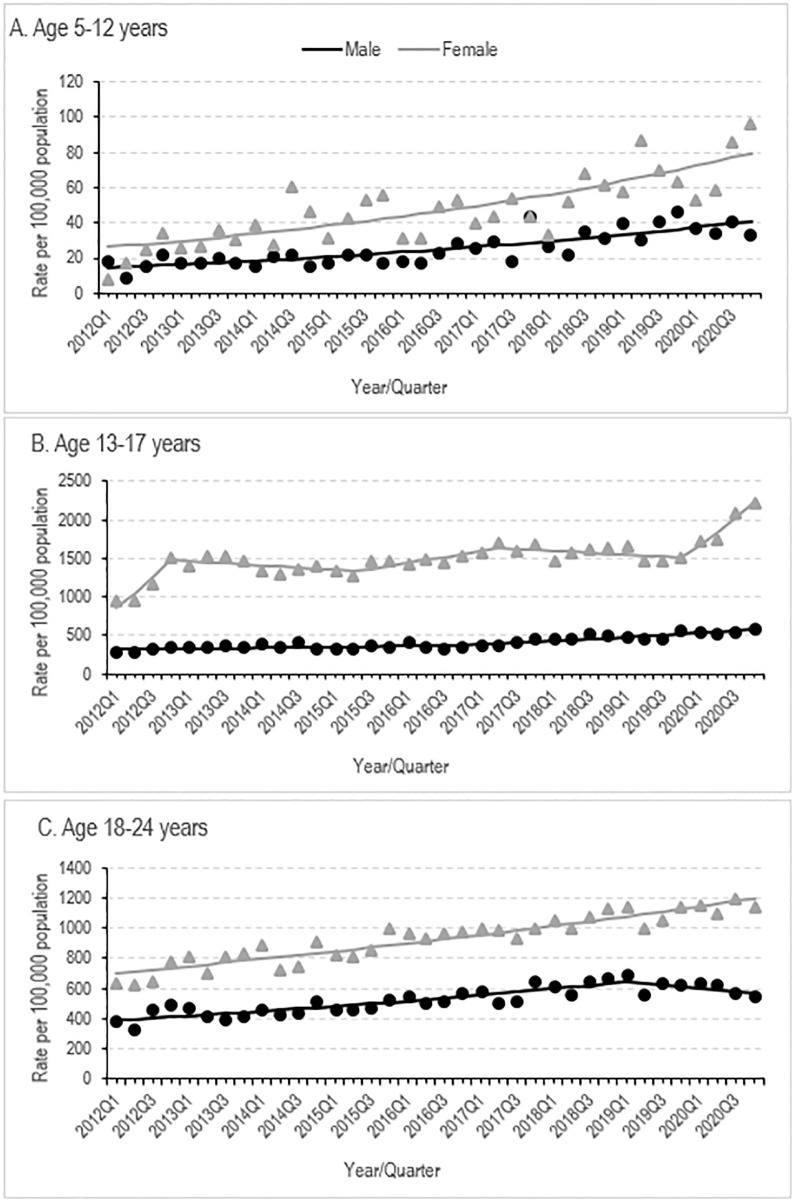
Emergency department self-harm presentations rates from 2012 to 2020 for males and females: Rates (symbols) and estimated trends (lines).

Between 2012 and 2020, long-term trends (AQPC) in hospital-presenting self-harm rates grew by an average of 2.0% per quarter (95%CI: 1.3–2.7%, *p* <0.001) for the total sample (AQPC: 1.6% males, *p* <0.001; AQPC: 2.4% females, *p* <0.001), with upward trends observed across all age groups. The largest average quarterly increases between 2012 and 2020 were observed in those aged 5–12 years in both females (3.2% per quarter) and males (3.0% per quarter) (Table 1, Fig 1). Among all other females, rates increased the most quarterly between 2012 and 2020 among aged 13–17 years (2.7% per quarter), followed by those aged 18–24 years (1.5% per quarter). Among all other males, rates increased by 1.7% per quarter in those aged 13–17 years, and 1.1% per quarter in those aged 18–24 years.

**Table 1:** Trends in ED self-harm presentation rates (per 100,000) by sex and age group among youth aged 5 to 24 years in New South Wales, for 2012 to 2020^.

Age Group (years)	Sex	APQC (95% CI)	Test Statistic (t), Prob > |t|	No. join points	QPC (95% CI)	Prob > |t|
**5–12**	Female	3.2 (2.4–4.1)	7.7, <0.001	0	-	-
	Male	3.0 (2.3–3.7)	8.8, <0.001	0	-	-
**13–17**	Female	2.7 (1.7–3.8)	5.2, <0.001	4	2012Q1–2012Q4: 19.4 (9.8–29.8) 2012Q4–2015Q2: -1.0 (-2.3–0.3) 2015Q2–2017Q2: 2.6 (0.7–4.5) 2017Q2–2019Q4: -0.8 (-2.0–0.5) 2019Q4 – 2020Q4: 10.3 (6.1–14.6)	<0.001, 0.114 0.010 0.209 <0.001
	Male	1.7 (1.1–2.4)	5.5, <0.001	1	2012Q1–2016Q4: 0.8 (0.0–1.7) 2016Q4–2020Q4: 2.8 (1.8–3.8)	0.062, <0.001
**18–24**	Female	1.5 (1.3–1.7)	14.1, <0.001	0	-	-
	Male	1.1 (0.5–1.8)	3.3, 0.002	1	2012Q1–2019Q1: 1.8 (1.4–2.2) 2019Q1- 2020Q4: -1.6 (-4.5–1.5)	<0.001, 0.301
**Total**	Female	2.4 (1.5–3.3)	5.2, <0.001	2	2012Q1–2012Q4: 12.3 (3.2–22.2) 2012Q4–2020Q1: 1.0 (0.8–1.2) 2020Q1–2020Q4: 6.4 (0.0–13.3)	0.009, <0.001, 0.050
	Male	1.6 (1.3–1.8)	13.0, <0.001	0	-	-

APQC: Average quarterly percentage change (describes the rate of change over the entire period); QPC: Quarterly Percent Change (describes the rate of change);

^ NSW emergency department self-harm presentation rate trends were calculated using joinpoint regression. The number and year/quarter of joinpoints associated with trends and determined statistically.

*5*–*12 years*

Based on multiple permutation tests that kept the overall level of type I error to less than 0.05, the final model selected zero join points for females and zero join points for males between 2012 and 2020. Between 2012Q1 and 2020Q4, rates in both females (3.2% per quarter) and males (3.0% per quarter) experienced significant and comparable increases.

*13*–*17 years*

Based on multiple permutation tests that kept the overall level of type I error to less than 0.05, the final model selected four join points for females and one joinpoint for males between 2012 and 2020. Between 2012Q1 and 2012Q4, the rate among females increased significantly by 19.4% per quarter and was subsequently followed by a nonsignificant quarterly decrease of 1.0% until 2015Q2. Starting in 2015Q2, the female rate increased significantly by 2.6% until 2017Q2, followed by a non-significant decrease of 0.8% until 2019Q4. Between 2019Q4 and 2020Q4, the female rate increased significantly by 10.3% per quarter. For males, after a non-significant increase of 0.8% per quarter between 2012Q1 and 2016Q4, the rate increased significantly by 2.8% per quarter until 2020Q4.

*18*–*24 years*

Based on multiple permutation tests that kept the overall level of type I error to less than 0.05, the final model selected zero join points for females and one joinpoint for males between 2012 and 2020. Starting in 2012Q1, the rate in females increased significantly by 1.5% per quarter until 2020Q4. In males, after a significant increase of 1.8% per quarter between 2012Q1 and 2019Q1, there was a non-significant decline of 1.6% per quarter until 2020Q4.

#### Time trends in urgency of ED self-harm presentations

**Fig 2:**
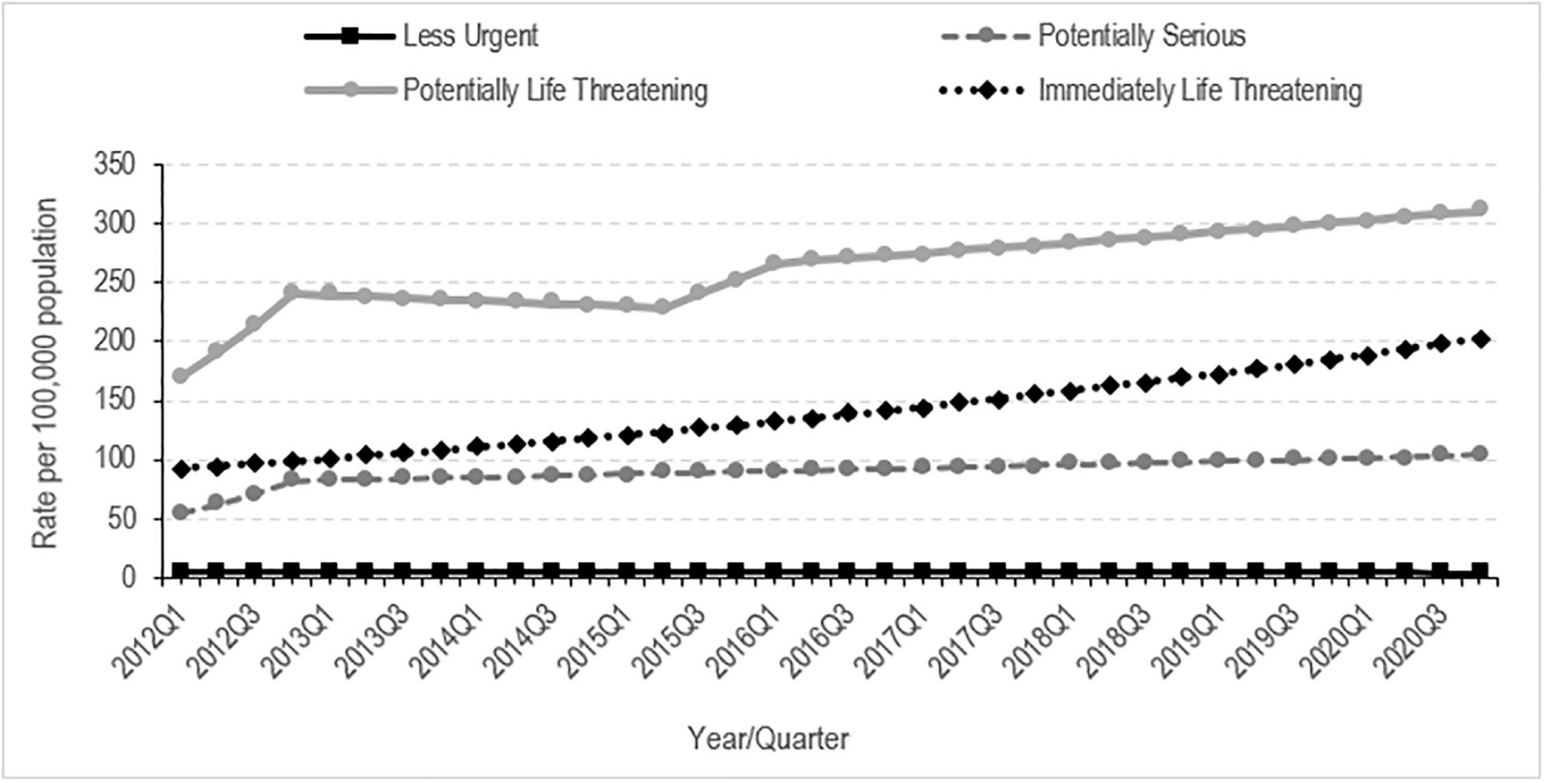
Emergence department self-harm presentations rates from 2012 to 2020 by triage category severity: Rates (symbols) and estimated trends (lines).

In the total sample, self-harm presentation rates by triage urgency were highest among the ‘potentially life-threatening’ category (261.2 rate per 100,000) followed by the ‘immediately life-threatening’ category (140.4 rate per 100,000) (Table 2). The greatest average quarterly growth by triage urgency was observed in the most serious ED presentation category (‘immediately life-threatening’), with an average quarterly percentage increase of 2.3% (Fig 2; S1 Table).

**Table 2:** Trends in ED self-harm presentation rates (per 100,000) among youth aged 5 to 24 years in New South Wales, 2012 to 2020.

	Females		Males		Total	
	Count	Rate	Count	Rate	Count	Rate
**Total**	58,039	710.5	25,072	292.7	83,111	496.7
**Age**						
5–12 years	1,529	46.8	867	25.1	2,396	35.6
13–17 years	28,941	1,498.5	8,135	399.8	37,076	934.8
18–24 years	27,569	929.0	16,070	522.6	43,639	722.2
**Country of birth**						
Australia	52,967	810.7	22,867	332.7	75,834	565.6
Other	5,072	311.4	2,205	130.8	7,277	219.1
**Remoteness**						
Major cities	37,629	608.4	16,439	253.7	54,068	426.9
Regional	19,821	1,023.6	8,194	402.4	28.015	705.2
Remote	305	819.4	168	436.0	473	624.4
Missing	284		271		555	
**Disadvantage (IRSD)**						
Most disadvantaged	13,122	763.1	5,950	326.2	19,072	538.2
Second most disadvantaged	13,294	787.8	5,439	306.4	18,733	541.0
Middle	12,169	744.4	5,264	308.8	17,433	522.0
Second least disadvantaged	9,784	775.5	4,176	317.9	13,960	542.0
Least disadvantaged	9,381	505.7	3,969	205.0	13,350	352.2
Missing	289		274		563	
**Mode of arrival (Ambulance arrival)**	28,170	344.9	13,820	161.4	41,990	250.9
**Admitted as in-patient**	16,072	196.8	6,522	76.1	22,594	135.0
**Triage category**						
Less urgent	561	6.9	286	3.3	847	5.1
Potentially serious	10,658	130.5	4,400	51.4	15,058	90.0
Potentially life-threatening	31,122	381.0	12,592	147.0	43,714	261.2
Immediately life-threatening	15,698	192.2	7,794	91.0	23,492	140.4
**Year**						
2012	4,750	523.4	2,155	226.4	6,905	371.4
2013	5,873	647.1	2,291	240.7	8,164	439.1
2014	5,733	631.7	2,465	259.0	8,198	440.9
2015	6,012	662.4	2,483	260.9	8,495	456.9
2016	6,449	710.6	2,686	282.2	9,135	491.3
2017	6,888	758.9	2,921	306.9	9,809	527.6
2018	7,049	776.7	3,288	345.5	10,337	556.0
2019	7,082	780.3	3,381	355.3	10,463	562.7
2020	8,203	903.8	3,402	357.5	11,605	624.2

IRSD: Index of relative Socio-economic Disadvantage; Remoteness and disadvantage measures (IRSD) based on person level area of residence at time of presentation.

The AQPC change by triage urgency was highest for the ‘immediately life-threatening’ category for all age groups, with an average increase of 3.5% for youth aged 5–12 years, an increase of 3.1% for youth aged 13–17 years, and an increase of 2.3% for youth aged 18–24 years (S2 Table). For the ‘potentially life-threatening’ and ‘potentially serious’ categories, youth aged 5–12 years reported the largest increases, followed by those aged 13–17 years and those aged 18–24 years (S2 Table).

#### Repeated ED presentations for self-harm

Approximately three-quarters of all persons who attended an ED for self-harm had no repeat presentations during the study period (74.5%, N = 38,141), while 22.0% (N = 11,259) presented between 2–4 times, and 3.5% (N = 1,781) had 5 or more separate repeat presentations. Persons aged 13–17 years at their index presentation were the group most likely to have re-presented to the ED for self-harm at any point (32.8%, N = 7,582), followed by persons aged 5–12 years (30.2%, N = 584), and 18–24 years (18.6%, N = 4,874). Among those who had a repeat presentation (n = 13,040 persons), 10.3% did so within one week (8.2% of 5–12 years; 9.0% of 13–17 years; 12.6% of 18–24 years) while 65.8% had re-presented within 12 months from the index presentation (57.0% of 5–12 years; 64.2% of 13–17 years; 69.3% of 18–24 years).

In the logistic regression analysis (Table 3), sociodemographic and ED presentation characteristics at the index self-harm presentation were potential predictive factors of repeated self-harm. Specifically, the odds of at least one re-presentation was significantly greater for those: aged 5–12 years (OR 1.87, 95%CI: 1.69–2.08) or 13–17 years (OR 1.98, 95%CI: 1.90–2.07), females (OR 1.56, 95%CI: 1.49–1.63), born in Australia (OR 1.43, 95%CI: 1.33–1.54), residing in a regional area (OR 1.05, 95%CI: 1.00–1.10), arriving by ambulance (OR 1.09, 95%CI: 1.05–1.14), being admitted as an in-patient (OR 1.19, 95%CI: 1.13–1.24), and having a triage category classified as potentially life-threatening (OR 1.06, 95%CI: 1.01–1.12).

**Table 3:** Binomial logistic regression for ED self-harm presentations.

	No re-presentation (single event) (N = 38,141 persons)	Multiple self-harm presentations (N = 13,040 persons)	Single versus multiple self-harm ED presentations
	N (%)	N (%)	OR (95% CI)
**Age group**			
5–12 years	1,351 (3.5)	584 (4.5)	1.87 (1.69–2.08)***
13–17 years	15,518 (40.7)	7582 (58.1)	1.98 (1.90–2.07)***
18–24 years	21,272 (55.8)	4874 (37.4)	1
**Female**	23,370 (61.3)	9,543 (73.2)	1.56 (1.49–1.63)***
**Australian born**	33,826 (88.7)	12,070 (92.6)	1.43 (1.33–1.54)***
**Remoteness**			
Major cities	25,206 (66.4)	8,267 (63.8)	1
Regional	12,483 (32.9)	4,619 (35.6)	1.05 (1.00–1.10)*
Remote	252 (0.7)	78 (0.6)	0.95 (0.73–1.23)
**Disadvantage (IRSD)**			
Least disadvantaged	8802 (23.2)	2,962 (22.9)	0.99 (0.93–1.06)
Second least disadvantaged	8302 (21.9)	2,910 (22.5)	0.99 (0.92–1.06)
Middle	8056 (21.2)	2,767 (21.3)	0.99 (0.92–1.06)
Second most disadvantaged	6437 (17.0)	2,154 (16.6)	0.98 (0.91–1.05)
Most disadvantaged	6336 (16.7)	2,171 (16.8)	1
**Ambulance arrival at index episode**	18,047 (47.3)	6,148 (47.2)	1.09 (1.05–1.14)***
**Admitted as in-patient at index episode**	9,549 (25.0)	3,564 (27.3)	1.19 (1.13–1.24)***
**Triage category at index episode**			
Less urgent	417 (1.1)	109 (0.8)	0.88 (0.71–1.10)
Potentially serious	7,048 (18.5)	2,382 (18.3)	1.01 (0.95–1.08)
Potentially life-threatening	20,076 (52.6)	7,227 (55.4)	1.06 (1.01–1.12)*
Immediately life-threatening	10,600 (27.8)	3,322 (25.5)	1

## Discussion

In this retrospective study we examined trends in hospital-presenting self-harm among young people aged 5–24 years for the period of 2012 to 2020, in NSW, Australia. A significant increase in youth hospital-presenting self-harm rates was observed across all age groups (2% per quarter) over the nine-year study period. These findings are in line with other studies showing that self-harm has been steadily rising over the past decade, e.g., [10,12,13], yet it remains unclear what is driving these trends. The greatest quarterly growth in the rates and severity of self-harm occurred among children aged 5–12 years (~3% per quarter). There were no clear differences in growth trends between girls and boys in this age group, contrary to findings reported elsewhere, where boys have been reported to have a higher risk of self-harming until around age 11 [19,20]. The highest rates of self-harm were observed in those aged 13–17 years (1,498.5 per 100,000), and females in this age group were almost four times more likely to present to hospital for self-harm than age-comparable males. The risk of repeat self-harm was nearly twice as high in children and adolescents as compared to young adults. As the risk of suicide death in young people with a history of self-harm is estimated to be approximately 30 times greater than in population-based controls [28,29], these findings highlight the need for better age-appropriate psychosocial assessment and early intervention, and improved access to effective treatments for self-harm following a hospital presentation.

The overall rate of hospital-presenting self-harm was 26.3 times higher in adolescents (13–17 years) than in children aged 5–12 years (934.8 versus 35.6 per 100 000) and 1.3 times higher than young adults, confirming that the risk for self-harm peaks in early-to-mid adolescence and substantially reduces with age [10,16,30,31]. This striking increase in self-harm behaviour between childhood and early adolescence (ages 12, 13) has also been observed in the few self-harm studies that have included data on children as young as 5 years of age [13,19,20]. This escalation in self-harm not only occurs alongside the onset of puberty, where difficulties in emotional control and risk-taking become more prominent, it is also timed to the transition from primary to secondary (high) school, a period of increased academic and interpersonal stress [1,4,32]. It is not surprising that alongside the spikes in self-harm from age 12, there are parallel increases in the onset of affective and mood disorders [33], both of which narrow young people’s ability to adaptively cope in stressful situations [34]. Unsurprisingly, mood disorders have been shown to confer the largest population attributable risk for self-harm in children and adolescents [35]. Given the strong concordance across many studies regarding the timing of self-harm trajectories, integrating proven effective interventions that target key risk processes (e.g., emotion regulation, problem solving) into settings of childhood (ages 0–12; e.g., primary schools), is an important goal for youth suicide prevention. In support of prevention approaches, there is high-quality evidence from a limited number of studies showing that prevention programs embedded in primary and secondary schools can reduce suicidal thoughts and behaviours by as much as 50% between exposure and control conditions [36].

Females across all age groups were over-represented in this study, presenting to hospital at rates that were between 1.78 and 3.75 times higher than age comparable males. Females aged 13–17 were a group of particular concern, experiencing some of the highest quarterly growth in self-harm of any age-group. For example, their self-harm presentation rates grew by approximately 20% in 2012, and subsequently by 10% from 2019 onwards. At the same time, self-harm rates only grew by between 0.8% and 2.8% in same-age males. Studies consistently shows that, across almost all life stages (except 5–11 years) females far exceed males in their rates of self-harm, and that adolescent females are largely responsible for the increases in self-harm rates that have been observed since 2011 [4,5,16]. Parallel increases in the rates of psychological distress, anxiety, and depression [37,38], all conditions that disproportionately affect females [39], have been posited as possible explanations for the increased risk of self-harm among females due to their negative impacts on coping. However, there are currently more questions than answers about what may be causing the distress underlying self-harm, and studies aimed at identifying and characterising risk processes among females are critically needed.

The overall repetition prevalence for self-harm in this study was 25%, making our findings consistent with other studies involving adolescents [9,40]. Importantly, our findings show that 10% of young people had re-presented to hospital for self-harm within *one week* of their index episode, while the majority (66%) had re-presented within the first 12-months. Other studies have also identified the first 12 months as a critical risk period for further self-harm and suicide [40], emphasising the need for hospitals to ensure there are processes in place to connect young people to appropriate and effective after-care services immediately upon discharge. Individuals aged 17 or younger and who were female had a significantly increased risk of self-harm repetition and may warrant particular attention during suicide risk formulation in hospital. Contrary to our findings, other studies have found no compelling evidence of sex-effects and have also found that older individuals are more prone to repeat self-harm [1,9,40]. Consideration of age-specific psychosocial assessments may be needed to meet the treatment needs of high-risk patients. In addition, living in regional or rural area of NSW was associated with greater odds of engaging in repeat self-harm. This increased risk may in part be attributable to shortages in mental health services and supports in rural or remote areas [41,42], which create a reliance on hospitals for care during a self-harm episode. Efforts at addressing social determinants of disadvantage and/or at reducing barriers to provision and access to mental health care in regional areas might confer reductions in the self-harm burden among young people.

### Strengths and limitations

While this study involves many self-harm events captured over a long duration, the ED administrative data was obtained from the NSW Health MHLL program, and as an externally linked dataset, we were unable to assess the accuracy of self-harm coding. Due to administrative coding restrictions in ED data, it was not possible to determine if self-harm events involved suicidal intent (suicide attempt) or non-suicidal intent. The study period and data we used covered the period 1 January 2012 to 31 December 2020. Index episodes were identified as the first record for an individual at any point during this period. However, individuals may have had self-harm episodes prior to 1 January 2012, and similarly, the true incidence of longer-term (e.g., 12-month) repeat presentations are likely to be underestimated for individuals with an index event occurring in 2020. Our data was only from a single State in Australia; a national analysis was not possible due to challenges in harmonising self-harm data across the different data capture systems used in the various States and Territories. While NSW is the most populous region of Australia with an estimated 8.5 million residents in 2022, of which 24% are young people aged 5–24 years [43], there may be differences in age, gender, and geography of young people which mean that our findings cannot be extrapolated to other States and Territories. Finally, the data used in this study overlaps with the early phase of the pandemic (March 2020 to December 2020) but we have not specifically examined impacts of COVID as such an analysis was recently conducted in another publication [16]. The specific impacts of COVID on quarterly growth trends is unknown in this study.

### Conclusion

Since 2012, self-harm presentations to the ED among children, adolescents, and young adults have been growing steadily in NSW, consistent with trends observed elsewhere in Australia and internationally. As self-harm can substantially increase the risk of suicide, identifying the reasons behind these sustained, upward increases is an urgent priority for the development of effective, developmentally appropriate strategies to support the mental health and wellbeing of young people.

## Supporting information

S1 Table Average trends in ED self-harm presentation rates (per 100,000) by triage urgency category among youth aged 5 to 24 years in New South Wales, 2012 to 2020^. (DOCX)

S2 Table Average trends in ED self-harm presentation rates (per 100,000) by triage urgency category and age group among youth aged 5 to 24 years in New South Wales, for 2012 to 2020^^^. (DOCX)
