# Supplementary material for: Self-harm in 5-to-24 year olds: Retrospective examination of hospital presentations to emergency departments in New South Wales, Australia, 2012 to 2020
Source: PLoS One. 2023 Aug 10;18(8):e0289877. doi: 10.1371/journal.pone.0289877 (PMC10414637; doi:10.1371/journal.pone.0289877)
Supplement: S1 Table — (DOCX) [file pone.0289877.s001.docx]

**S1 Table. Average trends in ED self-harm presentation rates (per 100,000) by triage urgency category among youth aged 5 to 24 years in New South Wales, 2012 to 2020^^^**

| **Triage Urgency Category** | **Period** | **Average Quarterly Percentage Change (95% CI)** | **Test Statistic (t)** | **Prob > \|t\|** |
| --- | --- | --- | --- | --- |
| Less urgent | 2012Q1-2020Q4 | -0.1 (-0.1 – 0.8) | -0.3 | 0.766 |
| Potentially serious | 2012Q1-2020Q4 | 1.9 (0.6 – 3.2) | 2.8 | 0.008 |
| Potentially life-threatening | 2012Q1-2020Q4 | 1.7 (0.6 – 2.9) | 2.9 | 0.007 |
| Immediately life-threatening | 2012Q1-2020Q4 | 2.3 (2.1 – 2.5) | 22.8 | <0.001 |

^^^ NSW emergency department self-harm presentation rate trends were calculated using joinpoint regression. The number and year/quarter of join points associated with trends and determined statistically. The average quarterly percentage change describes the rate of change over the entire period.
