# Supplementary material for: Self-harm in 5-to-24 year olds: Retrospective examination of hospital presentations to emergency departments in New South Wales, Australia, 2012 to 2020
Source: PLoS One. 2023 Aug 10;18(8):e0289877. doi: 10.1371/journal.pone.0289877 (PMC10414637; doi:10.1371/journal.pone.0289877)
Supplement: S2 Table — (DOCX) [file pone.0289877.s002.docx]

**S2 Table. Average trends in ED self-harm presentation rates (per 100,000) by triage urgency category and age group among youth aged 5 to 24 years in New South Wales, for 2012 to 2020^^^**

| **Triage Urgency Category** | **Age** | **Average Quarterly Percentage Change (95% CI)** | **Test Statistic (t)** | **Prob > \|t\|** |
| --- | --- | --- | --- | --- |
| **Less Urgent** | 5 – 12 years | - | - | - |
|  | 13 – 17 years | 0.3 (-0.9 – 1.5) | 0.6 | 0.552 |
|  | 18 – 24 years | -0.5 (-1.6 – 0.6) | -0.9 | 0.374 |
| **Potentially Serious** | 5 – 12 years | 3.1 (1.9 – 4.3) | 5.2 | <0.001 |
|  | 13 – 17 years | 2.5 (0.8 – 4.2) | 3.0 | 0.005 |
|  | 18 – 24 years | 1.8 (0.4 – 3.2) | 2.6 | 0.014 |
| **Potentially life-threatening** | 5 – 12 years | 3.1 (2.4 – 3.7) | 9.6 | <0.001 |
|  | 13 – 17 years | 2.4 (1.0 – 3.9) | 3.4 | 0.002 |
|  | 18 – 24 years | 0.9 (04 – 1.4) | 3.5 | 0.001 |
| **Immediately life-threatening** | 5 – 12 years | 3.5 (2.1 – 4.8) | 5.3 | <0.001 |
|  | 13 – 17 years | 3.1 (1.6 – 4.7) | 4.0 | <0.001 |
|  | 18 – 24 years | 2.3 (2.1 – 2.5) | 21.2 | <0.001 |

^^^ NSW emergency department self-harm presentation rate trends were calculated using joinpoint regression. The number and year/quarter of join points associated with trends and determined statistically. The average quarterly percentage change describes the rate of change over the entire period.
